# Proteomic analysis of exosomes secreted during the epithelial-mesenchymal transition and potential biomarkers of mesenchymal high-grade serous ovarian carcinoma

**DOI:** 10.1186/s13048-023-01304-0

**Published:** 2023-11-29

**Authors:** Germano Aguiar Ferreira, Carolina Hassibe Thomé, Clarice Izumi, Mariana Lopes Grassi, Guilherme Pauperio Lanfredi, Marcus Smolka, Vitor Marcel Faça, Francisco José Candido dos Reis

**Affiliations:** 1 Department of Gynecology and Obstetrics, Ribeirão Preto Medical School, Ribeirão Preto, SP Brazil; 2 Regional Blood Center of Ribeirão Preto, Ribeirão Preto Medical School, and Center for Cell Based Therapy, University of São Paulo, Ribeirão Preto, Brazil; 3 Department of Cellular and Molecular Biology and Pathogenic Bioagents, Ribeirão Preto Medical School, University of São Paulo, Ribeirão Preto, Brazil; 4 Department of Biochemistry and Immunology, Ribeirão Preto Medical School, University of São Paulo, Ribeirão Preto, Brazil; 5 Weill Institute for Cell and Molecular Biology, Cornell University, Ithaca, NY USA

**Keywords:** Ovarian cancer, Epithelial-mesenchymal transition, Extracellular vesicles, Exosomes, Secretome

## Abstract

**Background:**

The epithelial-mesenchymal transition (EMT) promotes cell signaling and morphology alterations, contributing to cancer progression. Exosomes, extracellular vesicles containing proteins involved in cell-cell communication, have emerged as a potential source of biomarkers for several diseases.

**Methods:**

Our aim was to assess the proteome content of exosomes secreted after EMT-induction to identify potential biomarkers for ovarian cancer classification. EMT was induced in the ovarian cancer cell line CAOV3 by treating it with EGF (10 ng/mL) for 96 h following 24 h of serum deprivation. Subsequently, exosomes were isolated from the supernatant using selective centrifugation after decellularization, and their characteristics were determined. The proteins present in the exosomes were extracted, identified, and quantified using Label-Free-Quantification (LFQ) via Liquid Chromatography-Tandem Mass Spectrometry (LC-MS/MS). To identify potential biomarkers, the obtained proteomic data was integrated with the TGGA database for mRNA expression using principal component analysis and a conditional inference tree.

**Results:**

The exosomes derived from CAOV3 cells exhibited similar diameter and morphology, measuring approximately 150 nm, regardless of whether they were subjected to EMT stimulation or not. The proteomic analysis of proteins from CAOV3-derived exosomes revealed significant differential regulation of 157 proteins, with 100 showing upregulation and 57 downregulation upon EMT induction. Further comparison of the upregulated proteins with the TCGA transcriptomic data identified PLAU, LAMB1, COL6A1, and TGFB1 as potential biomarkers of the mesenchymal HGSOC subtype.

**Conclusions:**

The induction of EMT, the isolation of exosomes, and the subsequent proteomic analysis highlight potential biomarkers for an aggressive ovarian cancer subtype. Further investigation into the role of these proteins is warranted to enhance our understanding of ovarian cancer outcomes.

**Supplementary Information:**

The online version contains supplementary material available at 10.1186/s13048-023-01304-0.

## Background

Ovarian cancer has the highest mortality rate among gynecological neoplasms. Of 314,000 women diagnosed worldwide in 2020, approximately 65% would die from the disease [1]. Among the various histotypes, high-grade serous ovarian carcinoma (HGSOC) is associated with the poorest prognosis [2].

Epithelial-mesenchymal transition (EMT) is a biological process associated with the development and progression of malignant tumors, involving cell motility and invasion, resistance to apoptotic stimuli, and acquisition of stemness. However, the precise connection between EMT and in vivo metastases remains unclear [3].

Exosomes, which are nanometer-sized vesicles, serve as mediators of intercellular communication by transferring proteins, genes, chemokine receptors, soluble factors, miRNAs, and other bioactive material. Tumor-derived extracellular vesicles are believed to contribute to the development and progression of cancer through various mechanisms, including the activation of hematopoietic and stromal cells, stimulation of angiogenesis [4], promotion of tumor dissemination, influence on the EMT process, remodeling of extracellular matrix, evasion of immune detection, and facilitation of the formation of pre-metastatic niches. Furthermore, exosomes hold promise as markers for the detection of metastases [5].

The discovery of proteins that regulate neoplastic growth and metastasis holds the potential to yield new biomarkers for early detection and prognosis of ovarian cancer, as well as targets for treatment. In this study, our objective was to identify potential biomarkers present in exosomes derived from ovarian cancer cells during the EMT process. We achieved this by employing mass spectrometry to identify proteins and comparing the results with publicly available gene expression data.

## Methods

### Ovarian cancer cell culture

CAOV3 (ATCC® HTB-75™) and SKOV3 (ATCC® HTB-77™) cells lines were cultured in Dulbecco’s modified Eagle medium (DMEM) (Gibco, Carlsbad, CA, USA) supplemented with 10% fetal bovine serum (FBS) (Thermo Scientific, Marietta, OH, USA). The OVCAR3 (ATCC® HTB-161™) cells were cultured in Roswell Park Memorial Institute Medium (RPMI-1640) (Gibco) supplemented with 20% Fetal Bovine Serum (FBS). Both media were further supplemented with 100 U/mL penicillin and 100 µg/mL streptomycin (Gibco). The cells were maintained at 37◦C in a humidified incubator in an atmosphere of 5% CO_2_. Before use, the cell lines were authenticated by short tandem repeat (STR) profiling and confirmed to be mycoplasma negative [6]. All cell lines were acquired from the American Type Culture Collection (ATCC, Gaithersburg, MD).

### EMT induction

The cell lines were initially seeded in the supplemented medium. After 24 h, the cell lines were washed twice with PBS, and the media was replaced with an FBS-free medium. Following additional 24-hour incubation, the cells were treated with an FBS-free medium containing 10 ng/mL Epidermal Growth Factor (EGF) (Cat#236-EG-200, R&D Systems, Minneapolis, Minnesota, USA). The EGF-containing medium was replenished every 24 h for a total duration of 96 h [6].

### Protein extraction

The cells were washed with PBS twice and subsequently disrupted in lysis buffer (Cat#9803, Cell Signaling, Danvers, MA, USA) using three sonication cycles. Each sonication cycle lasted for 5 min with cooled water in an ultrasonic bath (Unique, São Paulo, SP, Brazil). Following sonication, the cell lysate was centrifuged at 20,000×g for 30 min at 4◦C. The supernatant containing the proteins of interest was collected, and the protein concentration was determined using the Bradford method (Bio-Rad, Hercules, CA). Finally, the samples were stored at -80◦C.

### Western blotting

The proteins were separated by SDS–PAGE and then electrotransferred onto PVDF membranes (GE Lifesciences, Pittsburgh, PA, USA). Next, the membranes were incubated with blocking buffer (25 mM Tris-HCl (pH 7.5), 0.5 M NaCl, and 0.1% Tween-20) containing 5% non-fat dry milk. Primary antibodies were added to the membranes, followed by incubation with a secondary antibody, specifically horseradish peroxidase-conjugated goat anti-rabbit IgG (Cat#7074, Cell Signaling), as per the manufacturer’s instructions. The primary antibodies used are listed in Supplementary Table 1. The membranes were then developed using ECL Western Blotting detection reagents (GE Lifesciences) and images were captured using a CCD-Camera (ImageQuant LAS 4000 mini, Uppsala, Sweden). Dosimetric analyses were performed using ImageJ software. The values are presented as the EMT/CT ratio, normalized according to the constitutive protein glyceraldehyde 3-phosphate dehydrogenase (GAPDH). GAPDH was chosen as a loading control because it is involved in glycolysis, a fundamental metabolic pathway in most cells, and its expression levels tend to be relatively stable under many experimental conditions.

### PathScan EGFR signaling array kit

The PathScan EGFR signaling array kit (Cat#12622, Cell Signaling) was utilized, which contains fixed antibodies specific to phosphorylated proteins in a chemiluminescent sandwich immunoassay format. Experiments were conducted following the manufacturer’s instructions. Densitometric analyses of the obtained data were performed using the protein array analyzer plugin for ImageJ software.

### ROS detection by fluorescence assay

The production of reactive oxygen species (ROS) was assessed using the intracellular fluorogenic reagent CM-H2DCFDA (C6827, ThermoFisher Scientific) according to the manufacturer’s instructions. To serve as controls, cell lines were incubated with PMA (50 nM) for 1 h to induce ROS accumulation through PKC activation. For the experimental groups, CAOV3, SKOV3, and OVCAR3 (both CT and EGF-treated) cell lines were incubated with 5 µM CM-H2DCFDA for 1 h before analysis. The analysis was performed at 37 °C in a 5% CO_2_ atmosphere. ROS using a FACSCalibur cytometer (Becton-Dickinson), and the fluorescence was detected in the FL1/FL2 channel. The acquired data were analyzed using FlowJo software (Treestar, Inc).

### Mitochondrial membrane potential (MMP) by fluorescence assay

The assessment of mitochondrial membrane potential was conducted using intracellular tetramethylrhodamine ethyl ester perchlorate (TMRE) (#13296, Cell Signaling), following the manufacturer’s instructions. To establish control, cell lines were incubated with carbonylcyanide 3-chlorophenylhydrazone (CCCP) (50 µM) at 37 °C for 15 min to disrupt the mitochondrial membrane potential. For the experimental groups, CAOV3, SKOV3, and OVCAR3 (both CT and EGF-treated) cell lines were incubated with 200 nM of TMRE for 20 min before analysis. The analysis was performed at 37 °C in a 5% CO_2_ atmosphere using a Varioskan LUX Multimode Microplate Reader. The reader settings included excitation around 550 nm and emission around 580 nm.

### Isolation of exosomes

Following the EMT induction, the culture medium was collected and subjected to centrifugation at 300×g for 10 min, followed by an additional centrifugation step at 3000×g at 4^o^C for 30 min. The resulting supernatant was then filtered using a 0.22 μm syringe filter for decellularization. Subsequently, the filtered solution was concentrated to a minimum volume of approximately 8 mL using an Amicon ultrafiltration system with a 100-kDa cutoff (Millipore, Billerica, MA, USA). The exosomes were isolated and separated from the concentrated solution using the exoEasy Maxi kit (Qiagen; Valencia, CA) as per the manufacturer’s instructions. Once isolated and separated, the, collected exosomes were stored in a freezer − 80^o^C.

### Quantification of exosomes by nanoparticle tracking analysis

The size, distribution, and quantification of the exosomes obtained from independent experiments were assessed using nanoparticle tracking analysis (NTA) performed on a NanoSight NS300 system (Malvern Instruments, Malvern, United Kingdom). The NTA data were analyzed using NanoSight software (version 3.2.16) [7].

### Characterization of exosomes by transmission electron microscopy (TEM)

Freshly isolated exosomes were fixed in phosphate buffer containing 3% glutaraldehyde (v/v) and 4% paraformaldehyde (v/v) at 4^o^C for 2 h. After centrifugation at 16,500×g for 30 min, the exosomes were resuspended in PBS and applied onto Formvar/carbon-coated electron microscopy grids. The exosomes were then visualized by negative contrast using a transmission electron microscope JEM-100 CX II (Jeol) equipped with a digital camera Hamamatsu ORCA-HR at magnifications of 50000 ×, 100000 ×, and 200000 × [7].

### Extraction of proteins from exosomes

The proteins were extracted from exosomes by disrupting them using a lysis buffer (8 M urea, 150 mM Tris (pH 8.0), 0.5% Octyl β-D-glucopyranoside (O.G) (Cat#O9882, Sigma, St Louis, MO, USA), and protease and phosphatase inhibitors (MSSAFE, Sigma). The disruption process involved three sonication cycles, with each cycle lasting 5 min using cooled water in an ultrasonic bath (Unique, São Paulo, SP, Brazil). After sonication, the samples were centrifuged at 20,000×g for 30 min at 4◦C. The supernatant was collected and the protein concentration was determined by the BCA protein assay kit (Pierce Biotechnology, Rockford, IL, USA).

### Proteomic analysis

The exosome proteins (5 µg) were first reduced with DTT (1:1 mg/mg) for 5 min at 95^o^C and alkylated (5:1 mg/mg). The proteins were separated by SDS-PAGE using 4–20% Mini-PROTEAN TGX Precast Protein Gels, Cat # 4561093, Bio-Rad). After SDS-PAGE, each gel lane was divided into 4 equal-sized pieces, and in situ digestion was performed individually for each piece.

For each gel slice, SDS and dye were removed using NH_4_HCO_3_ (50 mM) containing 50% acetonitrile (ACN), followed by a wash with pure ACN. The slices were dried in SpeedVac (Savant) and then rehydrated with 20 µL of NH_4_HCO_3_ (100 mM) containing 0.6 µg of trypsin (Promega) for approximately 30 min. The gel slices were then covered with sufficient NH_4_HCO_3_ (100 mM) and maintained at 37^o^C for 16–18 h for digestion.

After digestion, the peptides were extracted from the gel using an ACN gradient (50%, 70%, and 100%) containing 0.1% formic acid. The extracted peptides were successively transferred to a clean microtube and dried using a SpeedVac (Thermo Scientific). The dried samples were resolubilized in ACN 5% containing 0.1% formic acid and desalted using C18 Zip Tips^™^ columns (Sulpelco, Sigma) following the manufacturer’s instructions.

For capillary liquid chromatography, an equivalent of 1 µg digested proteins was injected onto a column with dimensions of 25 cm length x 100 μm internal diameter of column). The peptides were separated using a linear gradient of ACN (5 to 35%) containing 0.1% formic acid over a period of 90 min at a flow rate of 250 nL/min. The chromatographic system was coupled to a high-resolution mass spectrometer, Q-Exactive HF (Thermo Scientific). The mass spectrometer was operated with a capillary voltage of 3.2 kV, a capillary temperature of 200 °C, a resolution of 100,000, and an FT-target value of 1,000,000.

The spectra were acquired in a dependent mode, selecting the 15 most abundant ions with a + 2 or + 3 charge state in the range of 400 to 1600 m/z for HCD (Higher-energy C-trap dissociation) fragmentation and MS/MS analysis. To avoid peptide sequencing redundancy, a 45-second exclusion window was applied.

### Proteomic data analysis

The six separate files generated from the LC-MS/MS analysis were processed using the Maxquant quantitative proteomics software tool [8]. This software was used to handle and process the data resulting in the identification and the relative label-free quantification (LFQ) of the detected proteins [9].

The search criteria used during the analysis were as follows: trypsin enzyme with a tolerance of two lost cleavages, mass error tolerance for precursor peptide was 6 ppm in the main search, mass tolerance of 20 ppm for fragment ions (MS/MS); and false positive rate (FDR) of 1% for both proteins and peptides.

The identified proteins were subjected to relative LFQ, where the normalized intensity profiles (LFQ intensity) were calculated. For paired comparisons, at least 1 peptide identified by MS/MS was required (LFQ minimum ratio count = 1). The normalized intensity values (LFQ intensity) obtained from the analysis were used for statistical analyses using Perseus software version 1.6.7.0 [10].

### Data visualization and gene ontology analysis

The list of quantified proteins that showed regulation (< 0.5 and > 1.9) from our large-scale proteomics study was subjected to further analysis using public databases and available free open-source software. For the identification, clustering, and visualization of protein-protein interaction networks, we utilized the database STRING (http://string-db.org/). This allowed us to explore the functional relationships and interactions among the identified proteins.

To gain insights into the biological processes and functions associated with the identified proteins, we performed a Gene Ontology (G.O.) analysis using FunRich [11]. FunRich enables us to annotate the proteins based on their involvement in specific biological processes, molecular functions, and cellular components. Additionally, we used FunRich to identify proteins known to be secreted by cell vesicles, providing us with valuable information about the potential role of exosomes in the context of our study [11].

### Integrative and statistical analysis

To prioritize target candidate proteins, we used gene expression data of 489 HGSOC available in The Cancer Genome Atlas (TCGA) database. The samples were clustered into the molecular subtypes immunoreactive (110), mesenchymal (108), proliferative (136), and differentiated (133) [12], The gene expression data was accessed using the cBioPortal repository (http://www.cbioportal.org/).

To integrate and analyze the data, we employed R 4.1.1 (The CRAN project, https://www.r-project.org/) software. We utilized the singular value decomposition for principal component analysis (PCA) to identify major sources of variation in the gene expression data. This allowed us to gain insights into the overall patterns and relationships among the samples based on their gene expression profiles.

Furthermore, we employed a conditional inference tree algorithm to identify the main potential biomarkers associated with the different molecular subtypes of HGSOC. This approach enabled us to uncover key proteins that could serve as promising biomarkers for distinguishing between the subtypes and potentially providing important clinical insights.

## Results

### EMT induction and characterization of exosomes

**Fig. 1:**
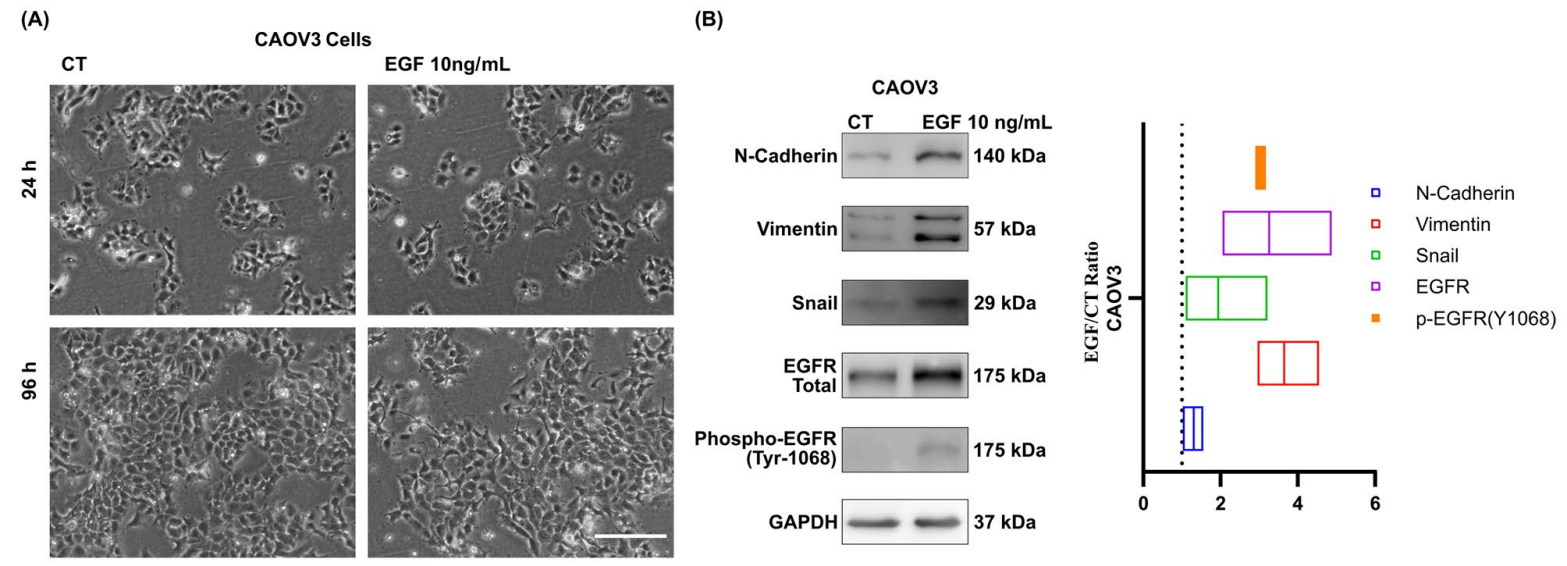
Characterization of EGF-induced Epithelial-Mesenchymal Transition (EMT). (**A**) Morphological changes observed by phase-contrast microscopy after 24/96 hours (Scale Bar – 200 μm). (**B**) Western blot analysis of EMT markers after 96 h of EGF-induced EMT in CAOV3 cells. The bar graph shows the EMT/CT ratio, a dosimetry measure, with CT representing control cells

Figure 1 shows the results of EMT induction. Following 96 h of EGF stimulation at 24-hour intervals, cells exhibited a spindle-shaped morphology and a loss of cell-cell contact (Fig. 1A). Western blot analysis of protein expression in CAOV3 cells illustrated the upregulation of N-cadherin, vimentin, SNAIL, total EGFR, and phospho-EGFR (Tyr1068) in cells induced to undergo epithelial-mesenchymal transition (EMT) by EGF treatment. The values in the graph represent the EMT/CT ratio, which is a densitometry measure, with CT representing control cells. This ratio has been normalized with respect to constitutive proteins, confirming the induction of EMT (Fig. 1B).

**Fig. 2:**
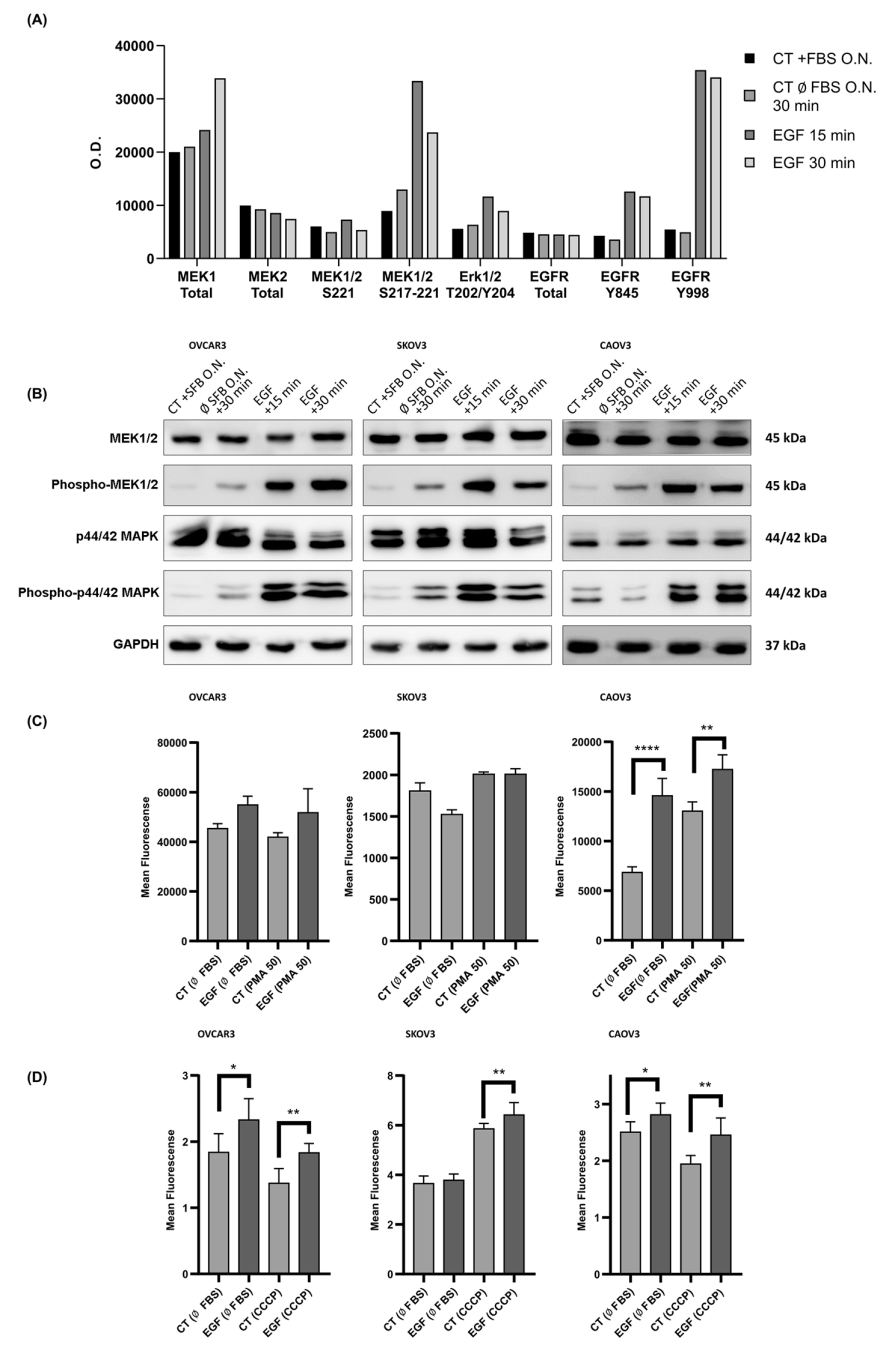
Characterization of EGF-stimulation. EGFR-signaling evaluation by (**A**) antibody-array (CAOV3 cells) and, (**B**) Western Blotting (OvCa cells) kept FBS-free for 18 h, followed by 15/30 minutes EGF-stimulation, showing MEK 1/2 total, phosphorylated MEK (Ser-217/Ser-221), p-44/42 MAPK (Erk 1/2) total, and phosphorylated p-44/42 MAPK (Erk 1/2) (Thr-202/Tyr-204). (**C**) evaluation of ROS (CM-H2DCFDA) and, (**D**) mitochondrial membrane potential (JC-1 aggregate/JC-1) of OvCa cells after EGF EMT-induction (mean ± SEM, *P < 0.05; **P < 0.01; ***P < 0.001; ****P < 0.0001)

We also observed an increase in the phosphorylation of EGFR residues Tyr845 and Tyr998, as well as, the effect of EMT induction on the phosphorylation of the downstream EGFR proteins MEK1/2 (Ser217 and Ser221) and p44/42 MAPK (Thr202 and Tyr204) in both OVCAR3 and SKOV3 cell lines. (Figure 2A and B). Moreover, cells undergoing EMT exhibited elevated levels of reactive oxygen species (ROS) and a decline in mitochondrial membrane potential (Fig. 2C and D).

**Fig. 3:**
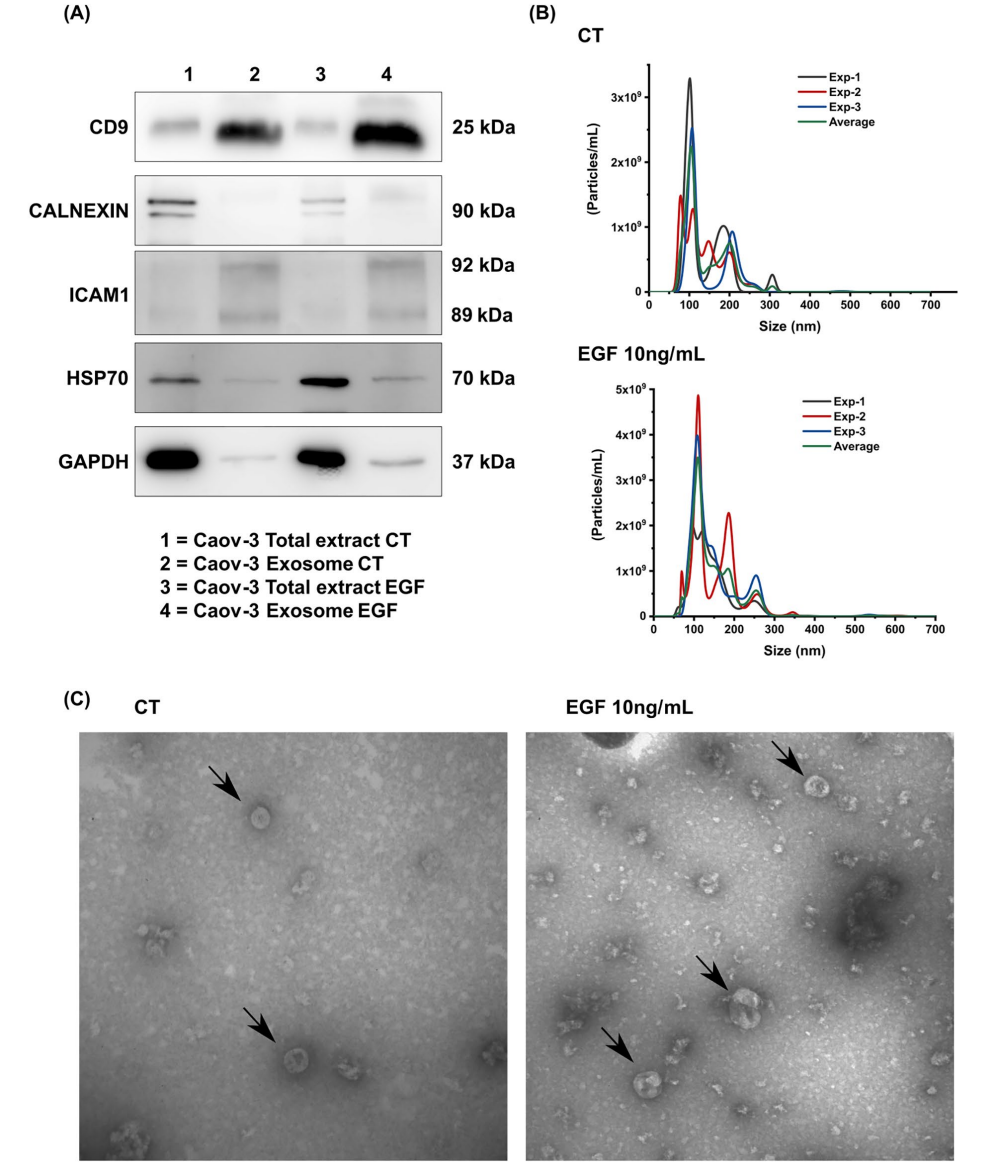
Characterization of exosomes induced by EGF-induced EMT. (**A**) Western blot analysis of exosome markers. (**B**) Evaluation of size and concentration using nanoparticle tracking analysis (NTA), revealing a mean size of 140.5 nm and a concentration of 1.24 × 10^9^/mL for the control (CT), and a mean size of 147.5 nm and a concentration of 2.18 × 10^9^/mL for EGF-induced EMT (EGF 10ng/mL). (**C**) Transmission electron microscopy (TEM) characterization of exosomes isolated from CAOV3 cells under control conditions (CT) and after 96 h of EGF-induced EMT (EGF 10ng/mL)

Figure 3 shows the exosome characterization. Protein extracts from exosomes exhibited a higher abundance of CD9 protein compared to protein extracts from the total cell lysate. The CD54-ICAM1 marker was exclusively identified in protein extract from exosomes, and the Hsp70 protein presented a lower amount in the protein extract from exosomes. The negative marker Calnexin was observed only in the total extract fraction (Fig. 3A) [13]. The diameter distribution was approximately 150 nm in both when comparing exosomes from EMT-induced and control cells. The exosome concentrations were 1.24 × 10^9^ for controls and 2.18 × 10^9^ for EMT-induced cells (Fig. 3B). Furthermore, the morphology of exosomes from EMT-induced cells and controls appeared similar under transmission electron microscopy (Fig. 3C).

### Discovery proteomics

We identified 1,898 proteins (FDR < 1%) in the exosomes secreted by CAOV3 cells. Among them, 157 proteins exhibited differential detection between exosomes from EMT-induced and control cells, with 57 proteins down accumulated and 100 proteins up accumulated (Supplementary Tables 2 and 3).

**Fig. 4:**
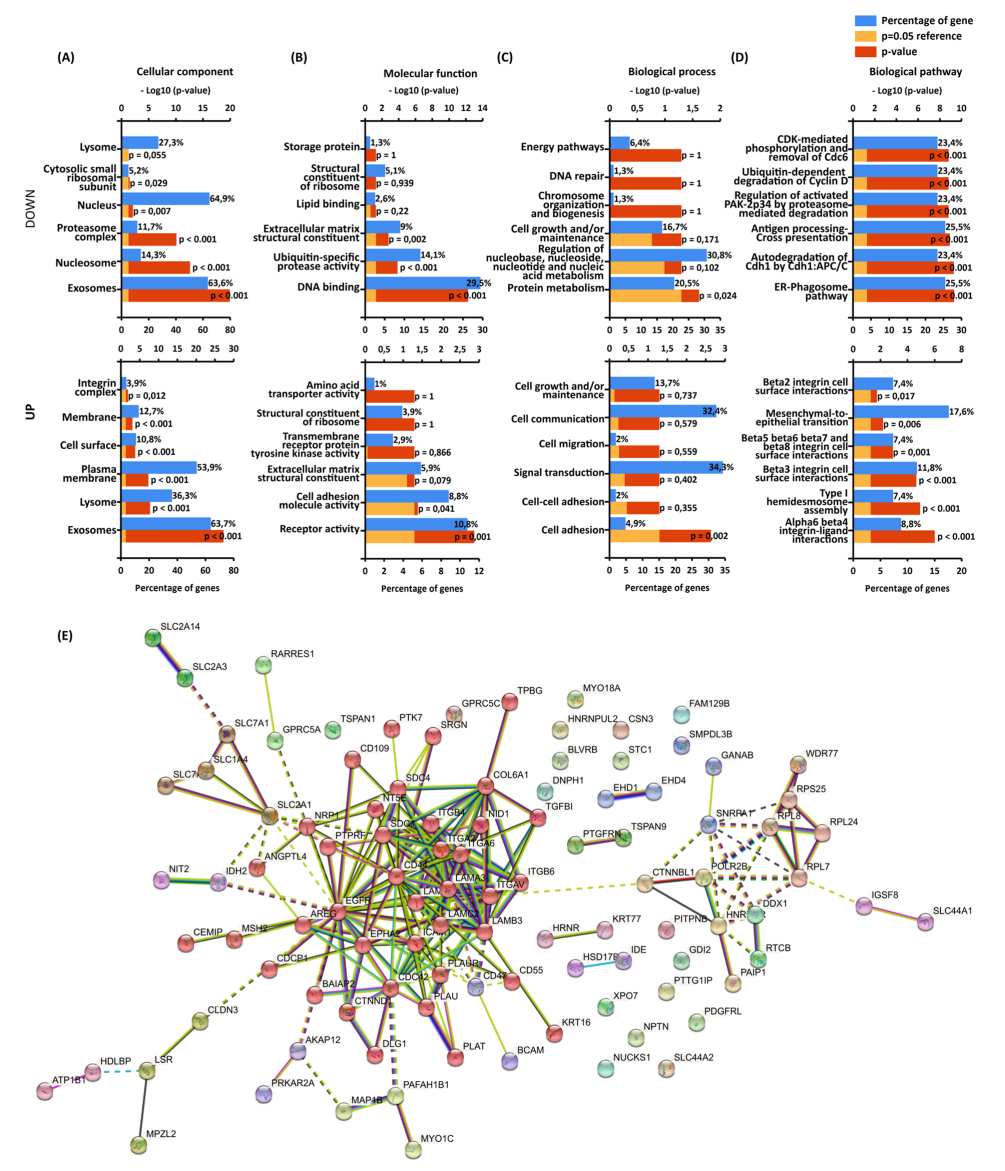
G.O., interaction, and network analysis of exosome-proteins identified by LC-MS/MS. G.O. analysis with FunRich software of exosome differentially regulated identified proteins (Down/Up): (**A**) Cellular component (CC), (**B**) Molecular Function, (**C**) Biological Process (**D**) and Biological Pathway. (**E**) Visualization and functional analysis of Protein-Protein Interaction Networks were performed using STRING (https://string-db.org/) MCL clustering

Figure 4 shows the Gene Ontology (G.O.) enrichment analysis of the list of differentially detected proteins. The exosomes carried proteins from several cellular components including lysosomes, the extracellular space, and the exosomes themselves (Fig. 4A). We identified proteins involved in multiple functional categories, such as the structural constituent of the extracellular matrix, DNA binding, ribosomal structural constituent, receptor activity, ubiquitin-specific protease activity, and lipid binding (Fig. 4B). These proteins were associated with various biological processes, including cell growth and maintenance, cell adhesion, protein metabolism, cell-cell adhesion, cell migration, and wound healing (Fig. 4C). Notably, EMT emerged as one of the major enriched biological pathways (Fig. 4D).

The protein-protein interaction networks of the 100 up-accumulated proteins from exosomes of EMT-induced cells revealed 19 clusters. The largest cluster, consisting of 39 nodes, exhibited strong associations with positive regulation of extracellular exosome assembly, extracellular matrix binding, and ECM-receptor interaction regulated by an EGF-like domain (Fig. 4E).

### Integrative analysis of proteomic data and TCGA mRNA expression

To identify potential classifiers of HGSOC subtypes, we integrated the list of 100 up-accumulated proteins identified from the exosomes of EGF-induced EMT cells with the results of HGSOC gene expression data based on molecular subtypes, including immunoreactive (110), mesenchymal (108), proliferative (136), and differentiated (133) [12]. We employed singular value decomposition for principal component analysis and utilized a conditional inference tree to identify key potential biomarkers.

**Fig. 5:**
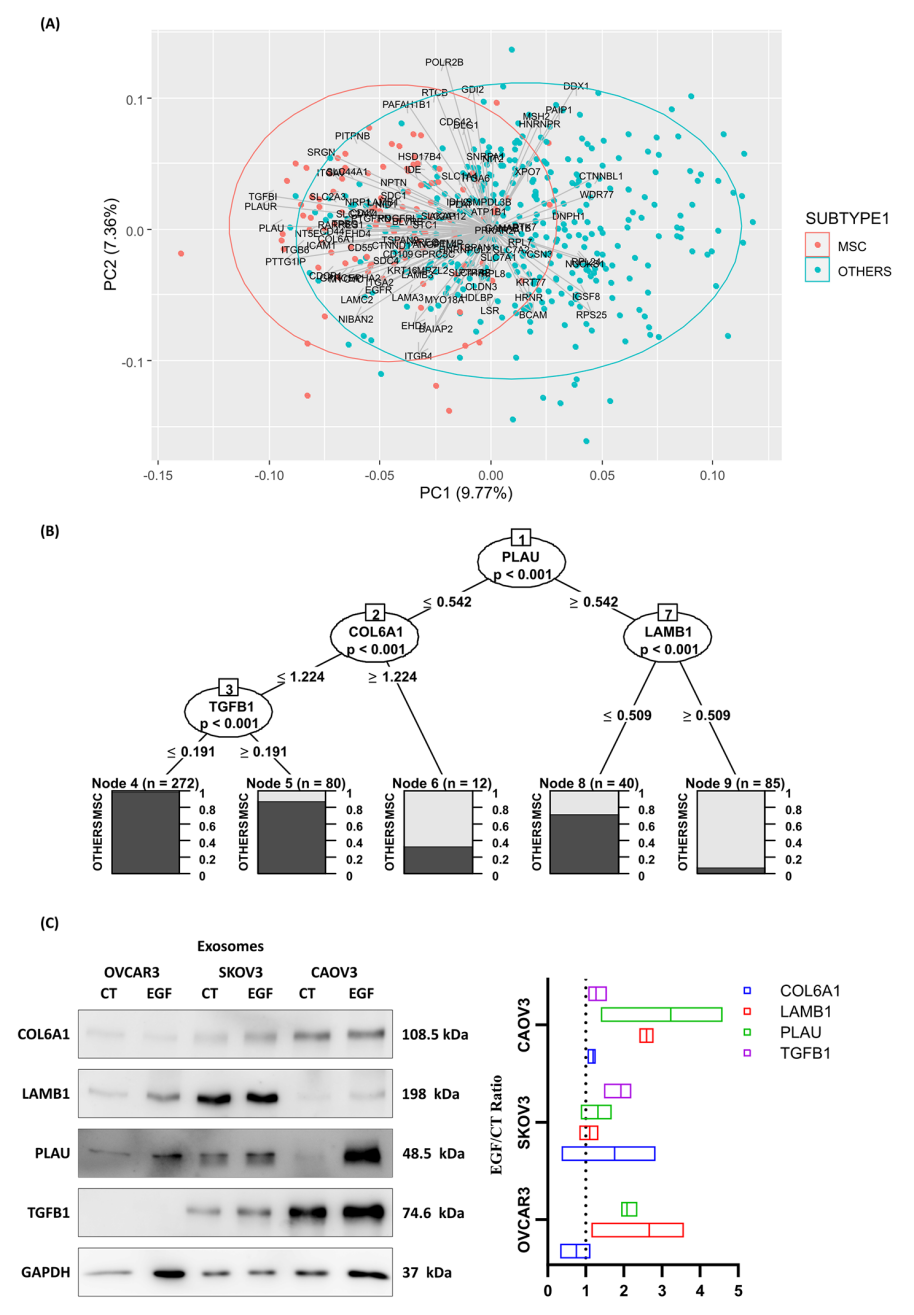
Correlation and prioritization of exosome-proteins identified by LC-MS/MS. Exosome proteins with an EGF/CT ratio > 1.9 were evaluated for their transcriptomic expression (z-Score) from the TCGA-OV cohort using (**A**) PCA followed by (**B**) Decision-Tree plotting, comparing the MSC subtype to the others. (**C**) Selected target exosome proteins COL6A1, LAMB1, PLAU, and TGFB1, were further evaluated by exosome western blotting. Bar graphs show EMT/CT ratio

Figure 5 A shows the principal component analysis of TCGA mRNA expression z-score of 100 genes associated with the up-accumulated proteins from exosomes of EMT-induced cell lines. We observed a distinct cluster representing the mesenchymal subtype HGSOC. Supplementary Table 4 shows the 39 genes with significantly higher expression in the mesenchymal subtype, as determined by univariate analysis.

Figure 5B shows a classification tree using TCGA mRNA z-score of 100 genes corresponding to up-accumulated proteins from exosomes of EMT-induced cell lines. The combination of the expression of the genes PLAU, LAMB1, COL6A1, and TGFBI classified correctly the majority of mesenchymal tumors. If PLAU presents a z-score > 0.54 and LAMB1 presents a z-score > 0.51, 91.8% of HGSOC were mesenchymal. If PLAU presents a z-score > 0.54 and LAMB1 presents a z-score ≤ 0.51, only 27.5% of HGSOC were classified as mesenchymal. When PLAU presents a z-score ≤ 0.54 and COL6A1 presents a z-score > 1.22, 66% of HGSOC were classified as mesenchymal. If PLAU presents a z-score ≤ 0.54, COL6A1 presents a z-score ≤ 1.22 and TGFB1 presents a z-score > 0.19, 12.5% of HGSOC were classified as mesenchymal. If PLAU presents a z-score ≤ 0.54, COL6A1 presents a z-score ≤ 1.22 and TGFB1 presents a z-score ≤ 0.19, 0.4% of HGSOC were classified as mesenchymal. The Western Blotting analysis proteins from SKOV3, OVCAR3, and CAOV3 cell lines confirmed the presence of COL6A1, LAMB1, PLAU, and TGFB1 in exosomes (Fig. 5C) with an increase after EGF stimulation.

## Discussion

Our study revealed that EMT induction enhances the concentration of exosomes secreted by ovarian cancer cells, and these exosomes carry potential tumor markers associated with the mesenchymal HGSOC subtype. Through evaluating changes in the proteome of exosomes derived from EGF-stimulated CAOV3 cells and integrating the data with the mRNA expression profile of the HGSOC from the TCGA-OV cohort, we identified PLAU, LAMB1, COL6A1, and TGFB1 as potential markers of mesenchymal HGSOC subtype.

The CAOV3 EMT induction model was previously developed by our research group to emulate key aspects of cancer progression and metastasis [6, 14]. The induced EMT phenotypes in this model include enhanced motility, invasiveness, and resistance to cisplatin which have been shown to arise from the stimulation of the ERK/MAPK signaling pathway based on mechanistic studies [15]. EGF stimulation plays a role in matrix remodeling through the ERK and ILK/GSK-3 pathways in ovarian surface epithelial cells [16], and the combined targeting of endothelin and EGF receptors has been shown to increase antitumor activity in ovarian cancer [6, 17]. Furthermore, the activation of NOX enzymes, some of which are located in the plasmatic membrane, triggers EGFR signaling, leading to increased H2O2 levels. This, in turn, results in sulphenylating Cys797 residues of EGFR, creating a positive feedback loop and amplifying kinase activity [18–20].

In a study by Grassi et al. [6] the cell proliferation rate was measured using BrdU incorporation, and it was demonstrated that the proliferation rate of Caov-3 cells remained unchanged after EGF-induced EMT. This finding indicated that proliferation does not directly influence the production and release of exosomes.

EMT potentially plays a significant role in the outcomes of HGSOC, which is a highly lethal gynecological malignancy [2]. EMT endows tumor cells with metastatic characteristics, enhancing their mobility, invasiveness, resistance to apoptosis, and stem cell-like properties [3]. The cancer cell secretome plays a crucial role in maintaining the tumor microenvironment, facilitating disease progression, and metastasis [21], with exosomes emerging as key players in this context [5]. Exosome-derived proteins from tumor cells mediate binding, motility, and degradation of the extracellular matrix, activation of hematopoietic cells and stroma, angiogenesis, and promotion of EMT in neighboring non-metastatic tumor cells [4].

Exosomes serve as carriers for a diverse range of molecules, including proteins, nucleic acids, lipids, and metabolites. In our study, among the differentially accumulated proteins in exosomes from EMT-induced cells and controls, a significant number belonged to lysosomes, nucleosomes, and proteasomes. The analysis of vesicles derived from tumor cells, as well as non-classical secretion processes, revealed intracellular proteins within extracellular vesicles [9, 22, 23]. Additionally, extracellular vesicles transmit EMT signals. Our study identified several binding-associated proteins, particularly involved in extracellular matrix (ECM) restructuring, cell growth, and motility and migration. These proteins may play a role in tumor EMT induction and metastasis. Overexpression or activation of these pathways in the tumor microenvironment can lead to increased resistance to treatment. The ECM of tumors is recognized as a significant barrier to effective cancer treatment [24, 25]. High expression of ECM remodeling genes distinguishes the mesenchymal molecular subtype from the other three HGSOC subtypes. The mesenchymal subtype is associated with upper abdominal and omental metastases, suboptimal surgical debulking, residual macroscopic disease after surgery, severe post-operative complications, and reduced overall survival [26]. Interactions between circulating tumor cells and the distant microenvironment can influence the formation of a metastatic lesion and colonization, contributing to the development of a metastatic niche through the extracellular matrix. In murine cancer models, combination therapies targeting global ECM deposition, such as TGF inhibitors, or specific components of ECM, such as in situ hyaluronic acid breakdown, have shown the potential to enhance responsiveness to therapy [6, 27–30].

The integrated analysis of EMT-induced exosome proteomic data and TCGA-OV cohort gene expression z-scores [12] identified PLAU, LAMB1, COL6A1, and TGFB1 as potential markers to classify mesenchymal HGSOC subtype. The PLAU gene encodes a secreted serine protease known as plasminogen activator urokinase. Plasminogen activator urokinase converts plasminogen to plasmin promoting the degradation of the basement membrane and facilitating tumor invasion and metastasis. The urokinase-type plasminogen activator system has been associated with aggressiveness and poor prognosis in various solid tumors, including ovarian cancer [31]. Ovarian cancer patients, especially those in the advanced stage, often exhibit increased serum levels of PLAU and PLAUR [32, 33], but while its elevated level is linked to shorter progression-free and overall survival, it does not represent a standalone prognostic indicator [34].

The gene LAMB1 encodes laminin subunit beta 1. Laminins play a role in cell differentiation, migration, adhesion, and tumor cell invasiveness [35],. LAMB1 has been identified in the EMT process of chemoresistant ovarian cancer cell lines, contributing to a more invasive phenotype [35, 36]. Overexpression of LAMA4, LAMB1, and LAMC1 mRNAs has been associated with lower overall survival (OS) and progression-free survival (PFS) in ovarian cancer [37].

In osteosarcoma and lung metastasis, COL6A1 is associated with a worse prognosis. Additionally, COL6A1 can be transported by exosomes and influence the differentiation of normal fibroblasts into cancer-associated fibroblasts. This process, driven by TGF/COL6A1 signaling, promotes cell metastasis, invasion, and migration [38]. In ovarian cancer cells, COL6A1 has been identified as an effector gene that promotes invasion and metastasis [39, 40]. The upregulation of COL6A1 and TGFB1 expression in a hypoxic microenvironment may enhance cell proliferation [41].

The role of TGFBI in cancer is ambiguous. Hypermethylation of the TGFB1 promoter has been associated with the suppression of TGFB1 expression [42]. TGFB1 is positively regulated by a feedback loop involving FXYD5, TGF β/SMADs signaling drives EMT during ovarian cancer progression [43]. Macrophage-produced TGFB1 supports an immunosuppressive microenvironment in ovarian cancer [44]. TGFB1 is also associated with pathogenesis [45], facilitating migration and invasion [46]. Furthermore, TGFB1 enhances autophagy and mitophagy through PI3K/Akt and Ras-Raf-MEK-ERK pathways in ovarian cancer [47].

While our study has provided valuable insights into the proteomic analysis of exosomes secreted during EMT in CAOV3 cells and the identification of potential biomarkers for mesenchymal HGSOC, we recognize the limitation of utilizing a single cell line in the first step of our investigation. Future studies should consider incorporating multiple cell lines in all steps of the study to enhance the generalizability of our findings and to validate the clinical relevance of these potential biomarkers.

## Conclusions

Proteomic analysis of exosome proteins from multiple cell lines offers great potential for advancing our understanding of ovarian cancer biomarkers. This approach provides valuable tools for characterizing biomarkers associated with the disease. In our study, we successfully identified and validated candidate biomarkers in EMT-induced TP53 mutant ovarian cancer cell lines, shedding light on their relevance in ovarian cancer progression. The integration of experimental induction of EMT, exosome purification, large-scale proteomic analysis, and integration with gene expression data from the TCGA database allowed us to identify potential markers of the mesenchymal subtype of HGSOC. The identified markers, including PLAU, LAMB1, COL6A1, and TGFB1, warrant further investigation regarding their roles in prognosis, as potential predictors of debulking, and as targets for new therapeutic drugs. Continued research in this field holds the potential to drive advancements in the diagnosis, prognosis, and treatment of ovarian cancer, ultimately leading to improved outcomes for patients.

### Electronic supplementary material

Below is the link to the electronic supplementary material.

Additional file 1: **Supplementary Table-1**: Sources and properties of antibodies used in the western blot-ting. **Supplementary Table-2**: Table containing the list of 57 down (≤ 0.5) accumulated proteins identified by the proteomic analysis of exosomes secreted by the ovarian cancer cell line CaOV3 during EMT. **Supplementary Table-3**: Table containing the list of 100 up (≥ 1.9) accumulated proteins identified by the proteomic analysis of exosomes secreted by the ovarian cancer cell line CaOV3 during EMT. **Supplementary Table 4**: Transcriptomic expression (z-Score) of up accumulated proteins with EGF/CTRL ratio ≥ 1.9 in the exosome-enriched fraction during EMT induction in CAOV3 cells, and with higher median expression in the mesenchymal subtype

## Data Availability

The mass spectrometry proteomics data have been deposited to the ProteomeXchange Consortium via the MassIVE (https://massive.ucsd.edu/ProteoSAFe/static/massive.jsp) partner repository with the dataset identifier (MSV000089521). Any additional information required to reanalyze the data reported in this paper is available from the lead contact upon request.
