# Supplementary material for: Proteomic analysis of exosomes secreted during the epithelial-mesenchymal transition and potential biomarkers of mesenchymal high-grade serous ovarian carcinoma
Source: J Ovarian Res. 2023 Nov 29;16:232. doi: 10.1186/s13048-023-01304-0 (PMC10685605; doi:10.1186/s13048-023-01304-0)
Supplement: Supplementary file 1 — Additional file 1: Supplementary Table-1: Sources and properties of antibodies used in the western blot-ting. Supplementary Table-2: Table containing the list of 57 down (≤ 0.5) accumulated proteins identified by the proteomic analysis of exosomes secreted by the ovarian cancer cell line CaOV3 during EMT. Supplementary Table-3: Table containing the list of 100 up (≥ 1.9) accumulated proteins identified by the proteomic analysis of exosomes secreted by the ovarian cancer cell line CaOV3 during EMT. Supplementary Table 4: Transcriptomic expression (z-Score) of up accumulated proteins with EGF/CTRL ratio ≥ 1.9 in the exosome-enriched fraction during EMT induction in CAOV3 cells, and with higher median expression in the mesenchymal subtype [file 13048_2023_1304_MOESM1_ESM.docx]

**Supplementary Table 1.** Sources and properties of antibodies used in the Western blotting.

|  | Antibody | Company | Catalog Number | Source | P/M | Mass (kDa) | Dilution |
| --- | --- | --- | --- | --- | --- | --- | --- |
| 1 | N-cadherin | Cell signaling | #13116 | Rabbit | M | 140 | 1:1000 |
| 2 | Calnexin | Cell signaling | #2679 | Rabbit | M | 90 | 1:1000 |
| 3 | CD54/ICAM-1 | Cell signaling | #67386 | Rabbit | M | 89, 92 | 1:1000 |
| 4 | CD9 | Cell signaling | #13174 | Rabbit | M | 22, 24, 35 | 1:1000 |
| 5 | EGFR | Cell signaling | #2646 | Rabbit | M | 175 | 1:1000 |
| 6 | phospho-EGFR (Tyr1068) | Cell signaling | #3777 | Rabbit | M | 175 | 1:500 |
| 7 | p44/42 MAPK (Erk1/2) | Cell signaling | #4695 | Rabbit | M | 42, 44 | 1:1000 |
| 8 | phospho-p44/42 MAPK (Erk1/2) (Thr202/Tyr204) | Cell signaling | #4376 | Rabbit | M | 42,44 | 1:1000 |
| 9 | MEK1/2 | Cell signaling | #8727 | Rabbit | M | 45 | 1:1000 |
| 10 | phospho-MEK1/2 (Ser217/Ser221) | Cell signaling | #9154 | Rabbit | M | 45 | 1:1000 |
| 11 | GAPDH | Cell signaling | #2118 | Rabbit | M | 37 | 1:1000 |
| 12 | Hsp70 | SBI System Bioscience | EXOAB-Hsp70A-1 | Rabbit | P | 70 | 1:500 |
| 13 | SNAIL | Cell signaling | # 3879 | Rabbit | M | 29 | 1:1000 |
| 14 | Vimentin | Cell signaling | #5741 | Rabbit | M | 57 | 1:1000 |
| 15 | PLAU | Sigma-Aldrich | HPA008719 | Rabbit | P | 48.5 | 0.2μg/mL |
| 16 | COL6A1 | Sigma-Aldrich | HPA019142 | Rabbit | P | 108.5 | 1:1000 |
| 17 | TGFBI | Sigma-Aldrich | HPA017019 | Rabbit | P | 74.6 | 0.2μg/mL |
| 18 | LAMB1 | Sigma-Aldrich | HPA004132 | Rabbit | P | 198 | 0.1μg/mL |

**Supplementary Table 2.** List of of 57 down (≤0.5) accumulated proteins identified by the proteomic analysis of exosomes secreted by ovarian cancer cell line CaOV3 during EMT.

| Gene names | Sequence coverage [%] | Mol. weight [kDa] | Score | Sequence coverage CTRL [%] | Sequence coverage EGF [%] | Intensity CTRL | Intensity EGF | LFQ intensity CTRL | LFQ intensity EGF | EGF/ CTRL |
| --- | --- | --- | --- | --- | --- | --- | --- | --- | --- | --- |
| APOE | 63.1 | 36.2 | 236.2 | 63.1 | 57.4 | 6.76E+09 | 3.86E+09 | 7.05E+09 | 3.28E+09 | 0.47 |
| B2M | 18.3 | 8.5 | 6.8 | 18.3 | 18.3 | 7.51E+07 | 5.09E+07 | 8.70E+07 | 3.89E+07 | 0.45 |
| B4GALT1 | 8.0 | 43.9 | 9.5 | 8.0 | 6.0 | 2.34E+07 | 1.72E+07 | 2.71E+07 | 1.34E+07 | 0.50 |
| COL12A1 | 34.4 | 333.2 | 323.3 | 34.4 | 13.7 | 2.70E+09 | 4.12E+08 | 2.65E+09 | 4.52E+08 | 0.17 |
| CPVL | 13.9 | 54.2 | 16.5 | 10.5 | 9.5 | 1.12E+08 | 5.92E+07 | 1.18E+08 | 5.32E+07 | 0.45 |
| CRISPLD1 | 39.4 | 35.1 | 23.5 | 39.4 | 6.7 | 7.45E+07 | 1.74E+07 | 6.43E+07 | 2.77E+07 | 0.43 |
| DKK3 | 26.4 | 40.0 | 35.0 | 26.4 | 24.2 | 4.96E+08 | 2.45E+08 | 5.25E+08 | 2.15E+08 | 0.41 |
| EEF1E1;EEF1E1-BLOC1S5 | 17.8 | 19.8 | 10.4 | 17.8 | 12.1 | 6.69E+07 | 2.94E+07 | 6.58E+07 | 3.06E+07 | 0.47 |
| EIF2B1 | 13.4 | 33.7 | 10.9 | 13.4 | 13.4 | 5.21E+07 | 2.67E+07 | 5.33E+07 | 2.54E+07 | 0.48 |
| FBLN1 | 34.1 | 78.3 | 118.6 | 34.1 | 13.5 | 4.15E+08 | 1.97E+08 | 4.12E+08 | 1.99E+08 | 0.48 |
| FTH1 | 49.2 | 21.2 | 29.5 | 49.2 | 49.2 | 4.82E+08 | 3.42E+08 | 5.98E+08 | 2.23E+08 | 0.37 |
| GAS6 | 13.4 | 74.9 | 21.3 | 13.4 | 2.7 | 3.13E+07 | 8.37E+06 | 2.73E+07 | 1.24E+07 | 0.45 |
| H3F3B;H3F3A;HIST2H3A;HIST3H3;HIST1H3A;H3F3C | 32.6 | 10.3 | 13.7 | 22.8 | 29.3 | 8.07E+09 | 2.71E+09 | 8.52E+09 | 2.26E+09 | 0.27 |
| HIST1H2AJ;HIST1H2AH;H2AFJ;HIST2H2AC;HIST2H2AA3;HIST1H2AD;HIST1H2AG;HIST1H2AA;H2AFX | 27.8 | 18.5 | 34.9 | 27.8 | 27.8 | 7.92E+09 | 4.22E+09 | 9.22E+09 | 2.84E+09 | 0.31 |
| HIST1H2BN;HIST1H2BL;HIST1H2BM;HIST1H2BH;HIST2H2BF;HIST1H2BC;HIST1H2BD;H2BFS;HIST1H2BK;HIST1H2BA | 38.6 | 18.8 | 47.9 | 38.6 | 38.6 | 1.51E+10 | 9.33E+09 | 1.86E+10 | 5.55E+09 | 0.30 |
| HIST1H4A | 68.9 | 11.4 | 82.3 | 68.9 | 66.0 | 5.98E+10 | 3.84E+10 | 6.21E+10 | 2.89E+10 | 0.47 |
| HMGB2 | 31.6 | 24.0 | 11.3 | 31.6 | 23.9 | 1.42E+09 | 4.59E+08 | 1.32E+09 | 5.59E+08 | 0.42 |
| IDUA | 15.5 | 72.7 | 39.1 | 15.5 | 13.6 | 1.66E+08 | 5.72E+07 | 1.54E+08 | 6.86E+07 | 0.44 |
| LTBP1 | 12.8 | 186.8 | 69.4 | 12.0 | 5.6 | 1.91E+08 | 5.81E+07 | 1.98E+08 | 5.16E+07 | 0.26 |
| LUC7L2 | 14.8 | 54.2 | 22.2 | 11.6 | 12.0 | 3.31E+07 | 2.60E+07 | 4.11E+07 | 1.80E+07 | 0.44 |
| MFAP5 | 34.2 | 9.4 | 11.8 | 34.2 | 34.2 | 1.74E+08 | 2.93E+07 | 1.46E+08 | 5.65E+07 | 0.39 |
| MX1 | 11.9 | 75.5 | 18.7 | 10.3 | 5.6 | 7.50E+07 | 1.82E+07 | 6.38E+07 | 2.93E+07 | 0.46 |
| MYL6 | 36.6 | 16.3 | 36.2 | 36.6 | 36.6 | 3.46E+08 | 2.30E+08 | 4.10E+08 | 1.57E+08 | 0.38 |
| NDNF | 33.5 | 64.7 | 54.2 | 33.5 | 16.5 | 5.54E+08 | 1.77E+08 | 5.11E+08 | 2.20E+08 | 0.43 |
| NEK9 | 3.6 | 107.2 | 6.1 | 2.0 | 3.6 | 1.91E+08 | 4.30E+07 | 1.78E+08 | 5.60E+07 | 0.31 |
| PRDX4 | 34.3 | 30.5 | 19.0 | 29.2 | 29.5 | 7.01E+07 | 4.76E+07 | 8.05E+07 | 3.72E+07 | 0.46 |
| PSAP | 37.8 | 58.4 | 76.1 | 35.9 | 29.8 | 1.01E+09 | 5.24E+08 | 1.06E+09 | 4.55E+08 | 0.43 |
| PSM8;PSMB8 | 23.6 | 30.4 | 17.4 | 23.6 | 10.1 | 2.77E+08 | 3.40E+07 | 2.61E+08 | 4.96E+07 | 0.19 |
| PSMA2 | 27.8 | 25.9 | 38.3 | 27.8 | 21.4 | 2.85E+08 | 5.57E+07 | 2.42E+08 | 7.85E+07 | 0.32 |
| PSMA3 | 29.8 | 28.4 | 38.3 | 29.8 | 29.8 | 2.73E+08 | 8.79E+07 | 2.44E+08 | 1.05E+08 | 0.43 |
| PSMA4 | 35.9 | 24.5 | 16.9 | 35.9 | 20.9 | 2.56E+08 | 6.11E+07 | 2.44E+08 | 7.27E+07 | 0.30 |
| PSMA5 | 46.9 | 26.4 | 53.6 | 46.9 | 46.5 | 6.93E+08 | 2.69E+08 | 6.37E+08 | 2.80E+08 | 0.44 |
| PSMA6 | 49.6 | 28.1 | 64.5 | 49.6 | 24.6 | 7.23E+08 | 2.49E+08 | 7.33E+08 | 2.39E+08 | 0.33 |
| PSMA7 | 56.9 | 27.9 | 79.2 | 56.9 | 26.2 | 7.64E+08 | 1.26E+08 | 6.86E+08 | 1.99E+08 | 0.29 |
| PSMB1 | 44.4 | 26.5 | 39.7 | 44.4 | 34.4 | 5.69E+08 | 1.52E+08 | 4.88E+08 | 1.89E+08 | 0.39 |
| PSMB3 | 26.3 | 22.9 | 14.0 | 26.3 | 18.5 | 2.22E+08 | 6.69E+07 | 1.94E+08 | 8.88E+07 | 0.46 |
| PSMB5 | 37.3 | 28.5 | 48.2 | 37.3 | 14.8 | 1.87E+08 | 2.84E+07 | 1.72E+08 | 4.42E+07 | 0.26 |
| PSMD8 | 29.7 | 19.8 | 16.6 | 29.7 | 29.7 | 2.17E+08 | 1.73E+08 | 2.68E+08 | 1.22E+08 | 0.46 |
| QPCT | 14.4 | 40.9 | 27.6 | 14.1 | 14.4 | 4.72E+08 | 3.18E+08 | 5.27E+08 | 2.62E+08 | 0.50 |
| REEP5 | 15.3 | 21.5 | 8.9 | 10.6 | 15.3 | 1.48E+08 | 8.06E+07 | 1.75E+08 | 5.43E+07 | 0.31 |
| RPS12 | 40.2 | 14.5 | 12.9 | 33.3 | 32.6 | 1.34E+08 | 1.05E+08 | 1.64E+08 | 7.41E+07 | 0.45 |
| RPS20 | 22.7 | 13.4 | 11.0 | 22.7 | 19.3 | 1.60E+08 | 4.76E+07 | 1.52E+08 | 5.52E+07 | 0.36 |
| RPS27;RPS27L | 28.6 | 9.5 | 6.0 | 28.6 | 28.6 | 5.95E+07 | 2.04E+07 | 4.74E+07 | 2.30E+07 | 0.49 |
| RPS5 | 24.0 | 22.4 | 13.9 | 24.0 | 20.0 | 4.56E+07 | 2.30E+07 | 4.58E+07 | 2.28E+07 | 0.50 |
| SDF4 | 27.6 | 41.8 | 39.2 | 27.6 | 21.5 | 2.21E+08 | 9.95E+07 | 2.37E+08 | 8.39E+07 | 0.35 |
| SF3B4 | 9.0 | 44.4 | 14.4 | 9.0 | 9.0 | 3.93E+07 | 1.61E+07 | 3.76E+07 | 1.78E+07 | 0.47 |
| SF3B5 | 46.5 | 10.1 | 11.8 | 46.5 | 32.6 | 1.57E+08 | 6.14E+07 | 1.56E+08 | 6.15E+07 | 0.39 |
| SHMT1 | 17.8 | 53.1 | 17.5 | 17.8 | 8.1 | 6.15E+07 | 1.67E+07 | 5.34E+07 | 2.48E+07 | 0.46 |
| SPARC | 43.2 | 34.6 | 80.2 | 43.2 | 40.6 | 1.19E+10 | 7.35E+09 | 1.25E+10 | 6.20E+09 | 0.50 |
| SPTAN1 | 48.2 | 284.5 | 323.3 | 48.2 | 39.0 | 4.41E+09 | 2.14E+09 | 4.34E+09 | 2.16E+09 | 0.50 |
| THBS1 | 15.0 | 129.4 | 71.4 | 14.2 | 11.5 | 3.53E+08 | 1.63E+08 | 3.50E+08 | 1.66E+08 | 0.47 |
| TPM3 | 69.6 | 26.4 | 75.3 | 68.3 | 62.6 | 1.95E+09 | 1.29E+09 | 2.17E+09 | 1.05E+09 | 0.48 |
| TPM4 | 62.1 | 28.5 | 55.4 | 62.1 | 48.0 | 3.45E+08 | 1.46E+08 | 3.57E+08 | 1.34E+08 | 0.38 |
| TUBB8 | 22.7 | 49.8 | 67.5 | 22.7 | 22.7 | 5.62E+08 | 5.39E+08 | 7.37E+08 | 3.64E+08 | 0.49 |
| VTN | 33.9 | 54.3 | 63.7 | 33.9 | 7.7 | 4.57E+08 | 1.04E+08 | 4.77E+08 | 7.83E+07 | 0.16 |
| YWHAB | 43.1 | 28.1 | 38.8 | 43.1 | 40.7 | 3.47E+08 | 1.50E+08 | 3.37E+08 | 1.60E+08 | 0.48 |
| YWHAG | 46.6 | 28.3 | 88.2 | 46.6 | 43.7 | 8.11E+08 | 4.20E+08 | 8.56E+08 | 3.64E+08 | 0.43 |

**Supplementary Table 3.** List of 100 up (≥1.9) accumulated proteins identified by the proteomic analysis of exosomes secreted by ovarian cancer cell line CaOV3 during EMT.

| Gene names | Sequence coverage [%] | Mol. weight [kDa] | Score | Sequence coverage CTRL [%] | Sequence coverage EGF [%] | Intensity CTRL | Intensity EGF | LFQ intensity CTRL | LFQ intensity EGF | EGF/ CTRL |
| --- | --- | --- | --- | --- | --- | --- | --- | --- | --- | --- |
| AKAP12 | 18.2 | 191.5 | 88.8 | 5.2 | 18.2 | 1.10E+01 | 3.80E+01 | 1.03E+08 | 3.02E+08 | 2.93 |
| ANGPTL4 | 18.5 | 45.2 | 15.3 | 12.3 | 13.5 | 5.00E+00 | 7.00E+00 | 2.15E+07 | 1.23E+08 | 5.73 |
| AREG | 20.8 | 30.3 | 58.8 | 16.8 | 20.8 | 6.00E+00 | 2.10E+01 | 4.72E+08 | 1.31E+09 | 2.77 |
| ATP1B1 | 20.8 | 35.1 | 22.9 | 11.9 | 17.2 | 7.00E+00 | 1.10E+01 | 5.61E+07 | 1.09E+08 | 1.95 |
| BAIAP2 | 13.7 | 61.4 | 16.9 | 5.6 | 13.7 | 3.00E+00 | 1.30E+01 | 3.61E+07 | 8.13E+07 | 2.25 |
| BCAM | 22.6 | 63.7 | 32.1 | 14.8 | 20.6 | 7.00E+00 | 1.30E+01 | 3.52E+07 | 7.97E+07 | 2.27 |
| BLVRB | 18.9 | 22.1 | 9.2 | 18.9 | 14.1 | 4.00E+00 | 4.00E+00 | 6.86E+06 | 1.65E+07 | 2.41 |
| CD109 | 10.9 | 161.7 | 68.6 | 10.2 | 10.1 | 1.70E+01 | 2.00E+01 | 9.22E+07 | 1.96E+08 | 2.12 |
| CD44 | 16.4 | 81.5 | 56.5 | 11.9 | 16.4 | 2.80E+01 | 5.50E+01 | 6.35E+09 | 1.49E+10 | 2.35 |
| CD47 | 15.3 | 20.3 | 6.1 | 15.3 | 10.4 | 7.00E+00 | 6.00E+00 | 8.85E+07 | 1.76E+08 | 1.99 |
| CD55 | 27.0 | 49.3 | 30.8 | 14.2 | 22.7 | 5.00E+00 | 2.70E+01 | 1.50E+08 | 4.55E+08 | 3.03 |
| CDC42 | 23.5 | 15.2 | 16.9 | 23.5 | 23.5 | 4.00E+00 | 7.00E+00 | 1.85E+07 | 5.83E+07 | 3.16 |
| CDCP1 | 15.3 | 92.9 | 28.8 | 7.5 | 15.3 | 3.00E+00 | 1.20E+01 | 6.79E+07 | 1.34E+08 | 1.98 |
| CEMIP | 30.1 | 153.0 | 121.9 | 7.9 | 30.1 | 8.00E+00 | 7.00E+01 | 2.32E+08 | 9.99E+08 | 4.30 |
| CLDN3 | 22.3 | 23.3 | 11.2 | 11.8 | 22.3 | 3.00E+00 | 8.00E+00 | 4.07E+07 | 9.04E+07 | 2.22 |
| COL6A1 | 7.9 | 108.3 | 19.4 | 4.1 | 7.9 | 4.00E+00 | 8.00E+00 | 4.39E+07 | 1.01E+08 | 2.29 |
| CSN3 | 45.1 | 20.3 | 16.6 | 24.2 | 45.1 | 3.00E+00 | 8.00E+00 | 2.46E+07 | 3.94E+08 | 16.02 |
| CTNNBL1 | 10.4 | 65.7 | 11.0 | 2.6 | 10.4 | 4.00E+00 | 7.00E+00 | 2.72E+07 | 7.28E+07 | 2.68 |
| CTNND1 | 20.0 | 104.9 | 76.6 | 13.2 | 20.0 | 2.00E+01 | 3.20E+01 | 1.23E+08 | 3.18E+08 | 2.58 |
| DDX1 | 16.1 | 73.9 | 23.9 | 16.1 | 10.8 | 1.00E+01 | 1.00E+01 | 3.79E+07 | 8.74E+07 | 2.31 |
| DKFZp566H1924;NPTN | 13.0 | 34.8 | 6.9 | 9.7 | 13.0 | 2.00E+00 | 6.00E+00 | 1.36E+07 | 5.91E+07 | 4.35 |
| DLG1 | 10.4 | 99.8 | 24.4 | 5.8 | 10.4 | 5.00E+00 | 1.30E+01 | 3.33E+07 | 8.72E+07 | 2.62 |
| DNPH1 | 20.6 | 25.9 | 11.3 | 20.6 | 16.0 | 6.00E+00 | 6.00E+00 | 6.51E+07 | 1.74E+08 | 2.67 |
| EGFR | 21.4 | 134.3 | 102.3 | 17.4 | 21.4 | 3.20E+01 | 5.10E+01 | 2.55E+08 | 5.02E+08 | 1.97 |
| EHD1 | 40.7 | 61.9 | 80.9 | 17.3 | 40.7 | 1.60E+01 | 4.30E+01 | 2.87E+08 | 6.59E+08 | 2.29 |
| EHD4 | 34.6 | 61.2 | 88.0 | 16.5 | 34.6 | 5.00E+00 | 2.70E+01 | 1.07E+08 | 3.29E+08 | 3.08 |
| EPHA2 | 15.5 | 108.3 | 54.1 | 3.5 | 15.5 | 2.00E+00 | 2.60E+01 | 6.19E+07 | 3.61E+08 | 5.83 |
| FAM129B | 8.6 | 84.1 | 13.1 | 6.4 | 5.8 | 2.00E+00 | 5.00E+00 | 1.42E+07 | 3.35E+07 | 2.37 |
| GANAB | 45.4 | 106.9 | 209.2 | 43.0 | 43.6 | 9.00E+01 | 1.12E+02 | 2.45E+09 | 4.83E+09 | 1.98 |
| GDI2 | 33.7 | 50.7 | 40.3 | 30.6 | 31.2 | 1.70E+01 | 2.40E+01 | 1.81E+08 | 3.53E+08 | 1.95 |
| GPRC5A | 23.2 | 40.3 | 72.3 | 22.4 | 23.2 | 1.50E+01 | 3.10E+01 | 7.09E+08 | 2.23E+09 | 3.15 |
| GPRC5C | 15.8 | 50.1 | 20.5 | 8.4 | 15.8 | 4.00E+00 | 1.70E+01 | 4.20E+07 | 2.02E+08 | 4.82 |
| HDLBP | 7.4 | 141.4 | 16.9 | 4.9 | 5.1 | 5.00E+00 | 9.00E+00 | 1.68E+07 | 4.36E+07 | 2.59 |
| HNRNPR | 12.8 | 70.9 | 9.4 | 11.2 | 8.5 | 4.00E+00 | 4.00E+00 | 1.15E+07 | 5.17E+07 | 4.51 |
| HNRNPUL2;HNRNPUL2-BSCL2 | 26.2 | 85.1 | 115.0 | 20.5 | 20.9 | 1.80E+01 | 2.40E+01 | 1.26E+08 | 2.71E+08 | 2.16 |
| HRNR | 7.0 | 282.4 | 11.6 | 2.5 | 7.0 | 2.00E+00 | 5.00E+00 | 3.88E+06 | 8.81E+06 | 2.27 |
| HSD17B4 | 12.9 | 76.8 | 12.1 | 8.9 | 7.5 | 3.00E+00 | 4.00E+00 | 1.54E+07 | 3.82E+07 | 2.47 |
| ICAM1 | 27.4 | 57.8 | 118.8 | 27.4 | 27.4 | 3.20E+01 | 4.40E+01 | 6.57E+08 | 1.54E+09 | 2.35 |
| IDE | 9.6 | 118.0 | 22.8 | 9.6 | 6.2 | 1.00E+01 | 9.00E+00 | 3.76E+07 | 9.53E+07 | 2.53 |
| IDH2 | 5.3 | 50.9 | 4.0 | 5.3 | 5.3 | 1.00E+00 | 2.00E+00 | 5.35E+06 | 1.10E+07 | 2.05 |
| IGSF8 | 22.7 | 65.0 | 47.7 | 10.3 | 21.0 | 8.00E+00 | 1.90E+01 | 6.26E+07 | 2.36E+08 | 3.78 |
| ITGA2 | 29.6 | 129.3 | 166.8 | 23.0 | 29.6 | 4.50E+01 | 8.60E+01 | 8.70E+08 | 2.66E+09 | 3.06 |
| ITGA6 | 17.1 | 126.6 | 54.8 | 5.5 | 17.1 | 6.00E+00 | 3.20E+01 | 1.03E+08 | 3.08E+08 | 2.98 |
| ITGAV | 9.7 | 116.0 | 28.7 | 4.0 | 9.7 | 8.00E+00 | 1.90E+01 | 6.45E+07 | 1.63E+08 | 2.52 |
| ITGB4 | 17.5 | 202.2 | 83.0 | 4.1 | 17.5 | 7.00E+00 | 4.80E+01 | 4.10E+07 | 4.03E+08 | 9.84 |
| ITGB6 | 10.9 | 81.5 | 19.3 | 3.1 | 10.9 | 6.00E+00 | 1.60E+01 | 8.02E+08 | 1.81E+09 | 2.26 |
| KRT16 | 38.1 | 51.3 | 38.3 | 33.0 | 38.1 | 7.00E+00 | 1.10E+01 | 3.55E+07 | 7.72E+07 | 2.17 |
| KRT77 | 9.2 | 61.8 | 8.2 | 7.4 | 9.2 | 2.00E+00 | 3.00E+00 | 6.48E+06 | 2.06E+07 | 3.18 |
| LAMA3 | 19.8 | 366.7 | 323.3 | 9.8 | 19.8 | 5.30E+01 | 1.37E+02 | 1.04E+09 | 3.51E+09 | 3.36 |
| LAMB1 | 38.0 | 200.5 | 323.3 | 33.8 | 37.1 | 1.34E+02 | 1.81E+02 | 2.97E+09 | 6.07E+09 | 2.04 |
| LAMB3 | 34.2 | 129.6 | 119.7 | 16.6 | 32.7 | 1.70E+01 | 4.00E+01 | 1.87E+08 | 4.56E+08 | 2.44 |
| LAMC2 | 42.8 | 131.0 | 281.4 | 22.9 | 42.8 | 4.60E+01 | 1.06E+02 | 5.96E+08 | 2.99E+09 | 5.02 |
| LSR | 21.8 | 66.2 | 58.8 | 15.8 | 20.6 | 1.50E+01 | 2.10E+01 | 1.43E+08 | 2.97E+08 | 2.08 |
| MAP1B | 16.0 | 270.6 | 108.4 | 7.2 | 16.0 | 2.10E+01 | 4.90E+01 | 1.51E+08 | 3.88E+08 | 2.57 |
| MPZL2 | 14.4 | 24.5 | 9.0 | 14.4 | 14.4 | 4.00E+00 | 8.00E+00 | 6.77E+07 | 1.41E+08 | 2.08 |
| MSH2 | 8.6 | 104.7 | 18.5 | 7.7 | 6.2 | 6.00E+00 | 9.00E+00 | 1.71E+07 | 4.66E+07 | 2.73 |
| MYO18A | 5.4 | 231.1 | 21.7 | 3.4 | 3.1 | 4.00E+00 | 8.00E+00 | 8.76E+06 | 2.99E+07 | 3.42 |
| MYO1C | 17.2 | 119.0 | 50.6 | 7.9 | 17.2 | 9,00E+00 | 2,50E+01 | 7,88E+07 | 1,54E+08 | 1.96 |
| NID1 | 48.0 | 136.4 | 323.3 | 41.8 | 45.5 | 9.90E+01 | 1.45E+02 | 4.77E+09 | 1.05E+10 | 2.20 |
| NIT2 | 19.2 | 30.6 | 18.2 | 19.2 | 13.8 | 6.00E+00 | 4.00E+00 | 1.45E+07 | 3.15E+07 | 2.18 |
| NRP1 | 23.2 | 101.3 | 88.1 | 12.3 | 22.3 | 1.70E+01 | 4.30E+01 | 4.48E+08 | 1.22E+09 | 2.71 |
| NT5E | 42.2 | 63.4 | 149.4 | 28.2 | 42.2 | 3.00E+01 | 6.60E+01 | 9.71E+08 | 2.96E+09 | 3.05 |
| NUCKS1 | 18.5 | 27.3 | 16.9 | 12.3 | 14.0 | 5.00E+00 | 6.00E+00 | 1.39E+07 | 3.38E+07 | 2.43 |
| PAFAH1B1 | 10.7 | 46.6 | 14.4 | 10.7 | 10.7 | 4.00E+00 | 5.00E+00 | 1.40E+07 | 2.95E+07 | 2.10 |
| PAIP1 | 14.8 | 53.5 | 11.8 | 14.8 | 6.7 | 5.00E+00 | 7.00E+00 | 2.09E+07 | 4.41E+07 | 2.11 |
| PDGFRL | 9.1 | 41.9 | 7.5 | 9.1 | 9.1 | 2.00E+00 | 2.00E+00 | 7.12E+06 | 1.43E+07 | 2.01 |
| PITPNB | 32.8 | 31.6 | 15.8 | 16.2 | 32.8 | 2.00E+00 | 6.00E+00 | 1.82E+07 | 3.91E+07 | 2.14 |
| PLAT | 18.0 | 62.9 | 36.6 | 6.2 | 18.0 | 3.00E+00 | 1.50E+01 | 1.97E+07 | 1.19E+08 | 6.06 |
| PLAU | 32.1 | 46.9 | 39.4 | 15.0 | 32.1 | 7.00E+00 | 2.80E+01 | 3.15E+08 | 6.26E+08 | 1.99 |
| PLAUR | 34.0 | 37.0 | 63.3 | 34.0 | 34.0 | 2.00E+01 | 2.40E+01 | 2.12E+08 | 4.77E+08 | 2.25 |
| POLR2B | 10.9 | 133.1 | 23.7 | 4.3 | 9.3 | 4.00E+00 | 1.10E+01 | 3.09E+07 | 9.49E+07 | 3.07 |
| PRKAR2A | 17.6 | 45.5 | 19.7 | 17.6 | 14.9 | 6.00E+00 | 6.00E+00 | 2.75E+07 | 8.15E+07 | 2.96 |
| PTGFRN | 33.2 | 98.6 | 149.4 | 23.2 | 31.9 | 2.80E+01 | 5.60E+01 | 4.36E+08 | 9.23E+08 | 2.12 |
| PTK7 | 14.9 | 118.4 | 40.3 | 4.0 | 14.9 | 5.00E+00 | 1.40E+01 | 3.20E+07 | 7.50E+07 | 2.34 |
| PTPRF | 8.2 | 212.9 | 32.8 | 3.4 | 8.2 | 9.00E+00 | 1.80E+01 | 5.40E+07 | 1.46E+08 | 2.71 |
| PTTG1IP | 18.9 | 18.3 | 8.3 | 18.9 | 18.9 | 2.00E+00 | 5.00E+00 | 1.14E+07 | 6.99E+07 | 6.11 |
| RARRES1 | 21.4 | 33.3 | 19.9 | 17.0 | 21.4 | 5.00E+00 | 1.20E+01 | 5.34E+07 | 1.78E+08 | 3.33 |
| RPL24 | 24.8 | 14.4 | 7.6 | 24.8 | 17.4 | 3.00E+00 | 2.00E+00 | 1.49E+07 | 3.91E+07 | 2.62 |
| RPL7 | 32.7 | 24.4 | 12.9 | 26.9 | 26.9 | 5.00E+00 | 8.00E+00 | 9.82E+07 | 2.38E+08 | 2.42 |
| RPL8 | 13.2 | 22.4 | 6.6 | 13.2 | 13.2 | 2.00E+00 | 4.00E+00 | 3.77E+06 | 1.24E+07 | 3.29 |
| RPS25 | 15.2 | 13.7 | 4.8 | 15.2 | 15.2 | 1.00E+00 | 2.00E+00 | 1.34E+07 | 4.65E+07 | 3.47 |
| RTCB | 27.5 | 55.2 | 39.0 | 23.4 | 14.1 | 8.00E+00 | 9.00E+00 | 7.88E+07 | 1.66E+08 | 2.10 |
| SDC1 | 10.1 | 17.8 | 5.8 | 10.1 | 10.1 | 5.00E+00 | 9.00E+00 | 5.76E+08 | 1.53E+09 | 2.66 |
| SDC4 | 25.3 | 21.6 | 28.7 | 25.3 | 19.2 | 1.10E+01 | 2.50E+01 | 5.93E+08 | 1.75E+09 | 2.96 |
| SLC1A4 | 11.5 | 55.7 | 15.4 | 9.4 | 11.5 | 2.00E+00 | 7.00E+00 | 4.19E+07 | 1.27E+08 | 3.04 |
| SLC2A1 | 14.4 | 54.1 | 30.7 | 10.4 | 14.4 | 1.20E+01 | 2.20E+01 | 3.89E+08 | 9.80E+08 | 2.52 |
| SLC2A3;SLC2A14 | 15.7 | 53.9 | 79.2 | 15.7 | 15.7 | 2.00E+01 | 3.70E+01 | 5.64E+08 | 1.25E+09 | 2.21 |
| SLC44A1 | 9.6 | 73.3 | 14.2 | 5.9 | 8.1 | 7.00E+00 | 7.00E+00 | 6.30E+07 | 1.30E+08 | 2.06 |
| SLC44A2 | 12.9 | 80.1 | 22.9 | 6.2 | 12.9 | 7.00E+00 | 1.40E+01 | 7.14E+07 | 1.82E+08 | 2.56 |
| SLC7A1;SLC7A2 | 3.8 | 67.6 | 16.5 | 3.8 | 3.8 | 3.00E+00 | 5.00E+00 | 2.53E+07 | 5.99E+07 | 2.37 |
| SMPDL3B | 16.0 | 45.3 | 11.9 | 8.6 | 16.0 | 3.00E+00 | 8.00E+00 | 1.54E+07 | 6.07E+07 | 3.94 |
| SNRPA1 | 14.1 | 15.8 | 4.2 | 14.1 | 14.1 | 4.00E+00 | 2.00E+00 | 8.17E+06 | 3.33E+07 | 4.07 |
| SRGN | 36.7 | 17.7 | 45.4 | 31.6 | 36.7 | 1.60E+01 | 2.90E+01 | 8.31E+08 | 1.79E+09 | 2.15 |
| STC1 | 32.8 | 27.6 | 35.5 | 22.7 | 32.8 | 7.00E+00 | 1.90E+01 | 1.41E+08 | 3.00E+08 | 2.13 |
| TGFBI | 31.6 | 74.7 | 72.6 | 25.5 | 27.1 | 2.90E+01 | 3.70E+01 | 3.95E+08 | 8.20E+08 | 2.08 |
| TPBG | 8.3 | 46.0 | 11.0 | 5.0 | 8.3 | 1.00E+00 | 8.00E+00 | 2.96E+07 | 8.83E+07 | 2.98 |
| TSPAN1 | 7.5 | 26.3 | 26.2 | 7.5 | 7.5 | 6.00E+00 | 1.00E+01 | 1.17E+08 | 2.38E+08 | 2.04 |
| TSPAN9 | 9.6 | 29.6 | 10.6 | 9.6 | 9.6 | 6.00E+00 | 6.00E+00 | 3.35E+07 | 1.15E+08 | 3.44 |
| WDR77 | 17.8 | 36.7 | 33.9 | 10.8 | 13.5 | 2.00E+00 | 5.00E+00 | 4.70E+07 | 9.35E+07 | 1.99 |
| XPO7 | 15.4 | 124.1 | 41.9 | 8.6 | 10.9 | 7.00E+00 | 1.10E+01 | 2.89E+07 | 7.20E+07 | 2.49 |

**Supplementary Table 4.** Transcriptomic expression (z-Score) of up accumulated proteins with EGF/CTRL ratio ≥ 1.9 in the exosome-enriched fraction during EMT induction in CAOV3 cells, and with higher median expression in the mesenchymal subtype.

|  |  | MSC (n=108) | | | | Other (n=331) | | | |  |
| --- | --- | --- | --- | --- | --- | --- | --- | --- | --- | --- |
|  | EGF/ CTRL | Median | SEM | Higher Value | Lower Value | Median | SEM | Higher Value | Lower Value | p-Value |
| AKAP12 | 2.93 | 0.326 | 0.069 | 2.501 | -1.700 | -0.173 | 0.048 | 2.567 | -2.322 | < 0.001 |
| ANGPTL4 | 5.73 | -0.063 | 0.095 | 3.382 | -1.440 | -0.452 | 0.048 | 5.099 | -1.687 | < 0.01 |
| CD109 | 2.12 | 0.291 | 0.067 | 2.000 | -1.104 | -0.163 | 0.054 | 2.821 | -2.476 | < 0.001 |
| CD44 | 2.35 | 0.276 | 0.072 | 2.273 | -1.237 | -0.258 | 0.056 | 3.904 | -2.429 | < 0.001 |
| CD55 | 3.03 | -0.044 | 0.085 | 3.510 | -1.422 | -0.191 | 0.051 | 2.895 | -2.857 | < 0.01 |
| CEMIP | 4.30 | 0.391 | 0.069 | 1.996 | -2.540 | -0.202 | 0.053 | 2.238 | -3.074 | < 0.001 |
| COL6A1 | 2.29 | 0.993 | 0.092 | 3.428 | -0.740 | -0.408 | 0.040 | 2.608 | -2.637 | < 0.001 |
| CTNND1 | 2.58 | 0.188 | 0.090 | 2.331 | -2.316 | -0.114 | 0.060 | 2.415 | -4.086 | < 0.001 |
| EHD4 | 3.08 | -0.091 | 0.077 | 2.109 | -2.364 | -0.633 | 0.055 | 2.340 | -3.924 | < 0.001 |
| EPHA2 | 5.83 | 0.343 | 0.094 | 3.979 | -1.572 | -0.400 | 0.049 | 3.050 | -2.759 | < 0.001 |
| GPRC5A | 3.15 | 0.149 | 0.104 | 3.644 | -1.842 | -0.208 | 0.052 | 2.543 | -2.046 | < 0.001 |
| ICAM1 | 2.35 | 0.396 | 0.084 | 2.737 | -1.054 | -0.175 | 0.055 | 3.085 | -2.481 | < 0.001 |
| ITGA2 | 3.06 | -0.109 | 0.093 | 2.735 | -1.856 | -0.603 | 0.052 | 3.240 | -2.621 | < 0.001 |
| ITGAV | 2.52 | 0.636 | 0.075 | 2.286 | -1.281 | 0.109 | 0.059 | 2.949 | -3.251 | < 0.001 |
| ITGB6 | 2.26 | 0.102 | 0.107 | 3.193 | -2.343 | -0.181 | 0.054 | 2.770 | -2.639 | < 0.001 |
| LAMB1 | 2.04 | 1.032 | 0.063 | 3.016 | -0.544 | -0.351 | 0.049 | 3.950 | -2.509 | < 0.001 |
| LAMC2 | 5.02 | 0.266 | 0.089 | 2.490 | -2.444 | -0.053 | 0.054 | 2.978 | -4.593 | < 0.001 |
| MAP1B | 2.57 | 0.037 | 0.072 | 1.901 | -1.455 | -0.476 | 0.052 | 2.982 | -2.597 | < 0.001 |
| MYO1C | 1.96 | -0.096 | 0.091 | 3.288 | -2.694 | -0.574 | 0.060 | 3.030 | -3.314 | < 0.001 |
| NID1 | 2.20 | 0.766 | 0.076 | 4.931 | -0.927 | -0.438 | 0.037 | 3.797 | -1.668 | < 0.001 |
| NRP1 | 2.71 | 0.500 | 0.076 | 2.877 | -1.111 | -0.371 | 0.051 | 3.491 | -2.499 | < 0.001 |
| NT5E | 3.05 | 0.890 | 0.063 | 2.915 | -0.510 | -0.454 | 0.045 | 3.123 | -1.921 | < 0.001 |
| PAFAH1B1 | 2.10 | -0.800 | 0.127 | 3.177 | -3.571 | -1.388 | 0.074 | 5.651 | -4.354 | < 0.001 |
| PDGFRL | 2.01 | 0.488 | 0.054 | 1.906 | -1.088 | -0.413 | 0.047 | 2.631 | -2.858 | < 0.001 |
| PLAT | 6.06 | 0.384 | 0.052 | 1.742 | -0.833 | -0.127 | 0.051 | 2.783 | -2.463 | < 0.001 |
| PLAU | 1.99 | 1.236 | 0.065 | 2.562 | -0.966 | -0.541 | 0.040 | 2.202 | -2.023 | < 0.001 |
| PLAUR | 2.25 | 0.638 | 0.078 | 3.536 | -0.989 | -0.382 | 0.052 | 2.412 | -2.603 | < 0.001 |
| PTGFRN | 2.12 | 0.622 | 0.089 | 3.261 | -1.928 | -0.082 | 0.061 | 6.559 | -3.349 | < 0.001 |
| PTK7 | 2.34 | 0.401 | 0.094 | 3.849 | -2.929 | -0.088 | 0.066 | 5.879 | -2.662 | < 0.01 |
| PTTG1IP | 6.11 | 0.320 | 0.087 | 2.728 | -1.627 | -0.458 | 0.070 | 4.025 | -5.139 | < 0.001 |
| RARRES1 | 3.33 | 0.529 | 0.081 | 3.289 | -1.617 | 0.147 | 0.057 | 2.990 | -2.072 | < 0.001 |
| SDC1 | 2.66 | 0.948 | 0.089 | 3.977 | -1.754 | -0.205 | 0.051 | 2.738 | -2.003 | < 0.001 |
| SLC2A1 | 2.52 | 0.513 | 0.107 | 4.127 | -3.899 | 0.057 | 0.054 | 4.177 | -2.952 | < 0.001 |
| SLC2A14 | 2.52 | 0.625 | 0.083 | 4.090 | -0.915 | -0.278 | 0.050 | 3.961 | -3.033 | < 0.001 |
| SLC2A3 | 2.52 | 0.594 | 0.103 | 4.040 | -0.820 | -0.361 | 0.048 | 3.940 | -1.927 | < 0.001 |
| SRGN | 2.15 | 0.548 | 0.075 | 2.138 | -1.671 | -0.192 | 0.054 | 2.485 | -3.595 | < 0.001 |
| TGFB1 | 2.08 | 0.984 | 0.062 | 2.845 | -0.185 | -0.391 | 0.047 | 3.079 | -3.046 | < 0.001 |
| TPBG | 2.98 | 0.384 | 0.053 | 1.726 | -1.348 | -0.072 | 0.054 | 2.565 | -3.172 | < 0.001 |
| TSPAN9 | 3.44 | 0.600 | 0.077 | 3.587 | -0.959 | 0.142 | 0.067 | 4.821 | -2.882 | < 0.001 |
